# A self-locking loop as an alternative to purse-string suture in colon anastomosis: a feasibility study

**DOI:** 10.1186/s13104-017-2412-4

**Published:** 2017-02-08

**Authors:** Odd V. Höglund, Oskar Maxon, Anders Grönberg

**Affiliations:** 1 Department of Clinical Sciences, Swedish University of Agricultural Sciences, Box 7054, 750 07 Uppsala, Sweden; 2 CarpoNovum AB, Olofsdalsvägen 10, 302 41 Halmstad, Sweden

**Keywords:** Circular staplers, CREX, Colorectal surgery, End-to-end stapling anastomosis

## Abstract

**Background:**

Colorectal cancer is common in humans where treatment involves surgical removal of the cancerous part of the intestines. In the anastomosis procedure a purse-string suture may be time consuming to perform. The aim was to replace the purse-string suture, to develop and test a self-locking loop for temporary sealing of the lumen in colon anastomosis.

**Methods:**

A new device, a flexible band with a locking mechanism was constructed, the I-Tie^®^. Small protrusions, designed for increased friction between device and tissue, were added to one side of the flexible band in order to enhance the grip at closure of the loop around tissue. The device was initially tested in vitro on pig intestines. In an in vivo study, the short-term implant was tested in a new suture-free method, CREX, and with traditional circular staplers for colonic anastomosis. Ten female pigs of approximately 50 kg were used in the in vivo test. The self-locking device was used for closure of the lumen around anvils in CREX (n = 5), and around anvil in traditional circular stapler anastomosis (n = 5). Two self-locking devices were used in each animal.

**Results:**

The self-locking device could close the lumen of colon around the anvil and trocar. Subjectively, the device achieved a tight closure of the colon and did not interfere with the anastomosis techniques.

**Conclusions:**

The technology was perceived as potentially timesaving and easy to use. We conclude the device may be an alternative to the traditional purse-string suture for temporary closure of the colon lumen in colon anastomosis.

## Background

Colorectal cancer is among the most common cancer types in humans and among those associated with the highest mortality in both men and women. The incidence varies between countries but is reported to be 46.3 per 100,000 people-years in Europe [1]. Surgical removal is the basis of treatment for colorectal cancer [2–4]. Other reasons for colorectal resection are inflammatory disease of the intestines or trauma.

The conventional methods for achieving anastomosis of the colon to colon or rectum consists of either suturing or stapling the resected intestinal ends together, with stapling currently being the most common technique. The use of circular staplers shorten duration of surgery compared to manual suturing [5].

After the colon is transected, the anvil of the stapler instrument is inserted into the open end of the bowel and the colon lumen is closed around the anvil shaft using a purse-string suture, manually or by use of a purse-string clamp [6, 7]. The bowel end can be sealed by a staple row, in which case the trocar is extended from the stapler device to pierce the tissue [8, 9]. Alternatively, the trocar is extended through the open end of the colon, after which the lumen is closed around the trocar using a purse-string suture [6, 7]. It may be technically challenging to insert a hand-sewn purse-string suture, especially deep in the pelvis [10].

A new suture-free method for anastomosis of the colon to colon or rectum was developed, CREX (Colorectal anastomosis; Rejoin the intestine and validate the anastomosis; Extract samples for analysis; X-ray through connected catheters) by CarpoNovum AB (Olofsdalsvägen 10, 302 41 Halmstad, Sweden). The methodology is in principal based on a device that locks the two intestinal ends together while the lumen remains open and allows passage of intestinal content. As the anastomosis heals, the short term implant will detach 7–10 days after surgery and follow feces out the natural way [11].

In the new CREX method, as well as in traditional stapled anastomosis, the colon lumen is temporarily sealed using a stapler or a purse-string suture. With the purpose to provide an easy to use and less time consuming alternative for the temporary sealing of the colon, a new method was developed, based on the principle of a flexible, self-locking loop. The aim of this study was to evaluate the feasibility of using this self-locking loop as an alternative to purse-string suture for temporary sealing of the colon in conjunction with two different techniques for colon anastomosis.

## Methods

### The self-locking loop

The I-Tie^®^ device, a flexible band with a case at one end containing a locking mechanism was constructed and patented [12] using computer aided design (Solidworks, Dassault Systèmes SolidWorks Corporation, Concord, USA). The other end of the band could be fed through the locking case where the locking mechanism allowed motion in one way only by locking into perforations of the band. The flexible band formed a self-locking loop, similar in construction to that of a cable tie or hose tie. Small protrusions were added to the side of the flexible band that was constructed to form the inside of the loop in order to enhance the tissue grip at closure of the loop around tissue. A steel mould was made for injection molding of the flexible band. The material used for injection molding was polyamide 6 (Prototal PDS AB, Tistelvägen 1, 531 71 Vinninga, Sweden).

### Ethical approval/consent to participate

The Uppsala Animal Ethics Committee, Sweden and Swedish Board of Agriculture approved the study (C402/2012 and 38-9492/12).

### Test in tissue—in vitro

Fresh, lukewarm pig intestines were collected from the local abattoir for use in a cadaver test. The fatty tissue was dissected to expose the colon. The colon was transected approximately 15 cm from the anus. The CREX anvil was inserted into the lumen. The self-locking loop was placed around the transected colon and the loop was tightened around the anvil shaft. The closure was visually inspected and the locked loop was cut off and removed for inspection of the compressed colonic tissue. This in vitro test was repeated ten times.

### Test in tissue—in vivo

The self-locking loop was tested in vivo in ten pigs of approximately 50 kg anaesthetized primarily for testing of the CREX methodology. In five pigs, the colon was anastomosed using the size 29 anastomotic ring of CREX and in five using a conventional circular stapler (Touchstone circular stapler size 29) with a transanal approach. The pigs were placed in dorsal recumbency and the abdomen was accessed through a midline incision. The descending colon was localized and clamped with two large forceps. The colon was then transected between the two forceps. Surrounding tissue was dissected to expose the serosal surface of the colon and allow for serosa–serosa apposition of the anastomosis.

In the five pigs where CREX was used, two self-locking loops were used in each pig, one on each side of the anastomosis. The self-locking loop was placed around the transected colon, the loop was tightened around the anvil shaft and compressed the intestine against the anvil shaft. Following tightening of the loop, colon tissue protruding beyond the closed loop was resected. Excess band protruding from the locking case was cut off. The anastomosis procedure was then completed according to the CREX methodology.

In the five pigs where conventional circular staplers were used, the stapler anvil was used in the same manner as described for CREX. The circular stapler was inserted rectally and the trocar was aligned with the transected end of the colon. The self-locking loop was placed around the transected colon and the loop was tightened around the tying area of the trocar. Colon tissue protruding beyond the closed loop was resected and excess band protruding from the locking case was cut off. The anastomosis was then completed with circular stapling according to established procedure.

## Results

**Fig. 1:**
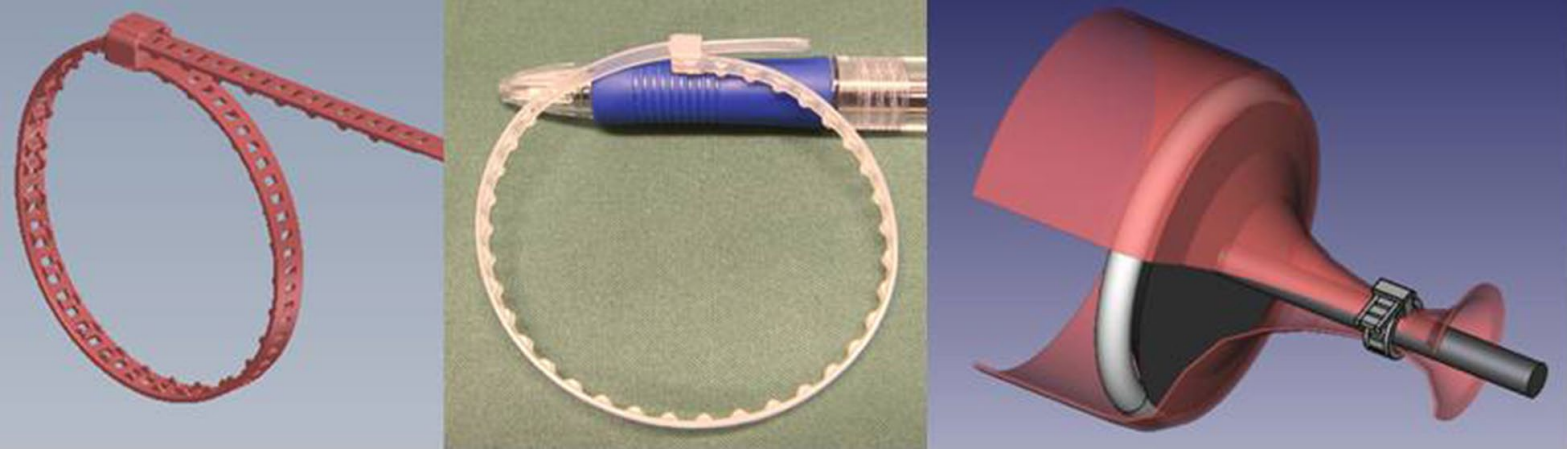
The constructed device formed a self-locking loop, the I-Tie^®^. Device shown with *ballpoint pen* for size reference. Width and height of the flexible band and locking case were 4 mm × 0.65 mm and 6 mm × 4.30 mm, respectively. *Right* drawing of the device placed around anvil, not corrected for relative size

The device was constructed and the product was injection molded. The device formed a self-locking loop (Fig. 1).

In the in vitro test the self-locking loop could close the colonic lumen around the CREX and stapler anvil in all 10 attempts.

Subjectively, the device achieved a tight closure of the colon lumen and a satisfying tissue grip. No damage to the colon tissue adjacent to the device was seen on visual inspection at removal of the self-locking loop. There were visual marks from compression of the colon tissue inside the tightened loop but no perforations were observed.

At removal of the loop it was noted that a small amount of tissue could be drawn into the locking case along with the flexible band as the loop was tightened. However, no interference with functionality of the device was observed.

**Fig. 2:**
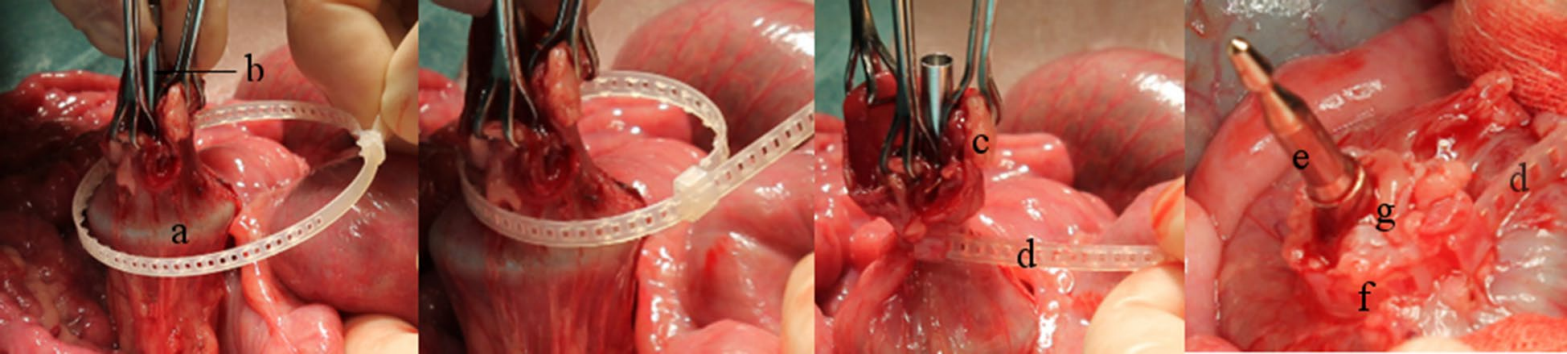
In the in vivo test the self-locking loop could close the colonic lumen around the anvil of both CREX and circular stapler as well as around the trocar of the circular stapler. **a** The CREX O-ring on anvil inside bowel. **b** The CREX anvil shaft. **c** Excess tissue protruding beyond tightened loop, to be cut off. **d** Excess band protruding from the locking case of the tightened loop, to be cut off. **e** Trocar of circular stapler. **f** Loop tightened around the trocar. **g** Colon held in place by the implant after removal of excess tissue protruding from the tightened loop

In the in vivo test the self-locking loop could close the colonic lumen around the CREX anvil (n = 10), the circular stapler anvil (n = 5) and the circular stapler trocar (n = 5), (Fig. 2). The device achieved a subjectively good tissue grip in all attempts. After resection of the tissue protruding beyond the closed loop, the compressed colonic tissue was firmly held in place by the closed loop in all attempts. Following cutting off the excess band protruding from the locking case, the anastomosis procedure could be continued without interference of the tightened device in place in both the CREX method and circular stapler anastomosis.

## Discussion

End-to-end stapling anastomosis of the intestines was developed in Russia, thereafter described in western literature in mid 70-ies [13] and further developed in the US [14]. CREX is a new method under development for anastomosis of the colon. In CREX as well as in the traditionally used method of circular stapling, the intestine is sealed using a stapler or a purse-string suture. The self-locking loop of the present study was tested as an alternative technique for closure of the colon lumen around the anvil shaft of CREX both in vitro and in vivo and additionally around the anvil and trocar of the circular stapler in vivo. This study demonstrated feasibility of using the self-locking device to close the lumen around the trocar and anvil that are used for colon anastomosis. The device may be an alternative to the purse-string suture, an adjuvant short-term implant of the surgeons’ armamentarium.

In the in vitro tests, the colon tissue was visually inspected after removal of the tightened loop. There were visual marks of compression of the colon tissue inside the tightened loop, but no perforation or other damage was observed. Even if perforating damage to the colon tissue inside the tightened loop could potentially occur, it may be considered clinically irrelevant as this compression is temporary and the loop along with this tissue will subsequently be removed at completion of the anastomosis. It was noted in the in vitro tests that a small amount of tissue could be pulled into the locking case along with the flexible band as the loop was tightened. This did not appear to cause any complications in this pilot study, but the possible hazard of tissue interfering with the locking mechanism was recognized and should be considered in future tests.

This study mostly tested the use of the self-locking loop in the conjuncture with the CREX methodology, as well as a few tests with the circular stapler device. However, owing to the similarities in the tying areas of the respective anvils and trocars of the devices, we suggest the possibility to extrapolate the results from one to the other.

Subjectively, the self-locking loop achieved a tight closure of the colon lumen and a satisfying tissue grip. In this feasibility study duration of surgery was not studied in detail but it was the subjective notion of the experienced surgeon conducting the in vivo tests that the self-locking loop would be time saving. There are several studies where self-locking loops, traditional cable ties, enabled an easier surgical procedure [15–18]. However, to test a hypothesis of an enabled shortened duration of surgery, further studies are needed.

In all tests, the self-locking loop was subjectively easy to use and easy to learn how to use. However, in the testing of the self-locking loop, some issues for design development were identified. A reduction of thickness of the tip of the flexible band may facilitate an easier introduction into the locking case. This measure could possibly be combined with enlarged tracks inside the locking case. A more rounded off design of the ventral aspects of the locking case may reduce the risk imposed by sharp edges. Additionally, it was suggested to add ridges to the outside of the locking case to enable a secure grip with forceps. In the testing described here, the loop was mostly tightened by hand. Depending on circumstances such as hard-to-reach anastomosis, laparoscopic surgery or the risk of hands slipping if the device or gloves get wet, a suggested improvement was that the band could be held and tightened by the use of forceps or other instruments. For this purpose, a gripping handle of sorts could be added to the locking case, enabling a secure fixation of the locking case inside the jaws of a pair of forceps. The described self-locking device was in principle similar to a cable tie or hose tie. For conventional cable ties, combined tools for tightening and cutting of the tie are available. The construction and use of a similar instrument for tightening and cutting of this device for laparoscopic surgery should be considered.

It was noted that for the self-locking loop to be usable with the smaller sizes of the surgical devices, such as the size 26 CREX, it is important that the circumference of the loop, fully tightened around tissue, remain sufficiently small to fit in the device housing. The size of the band is also of importance when it comes to potential laparoscopic use, where it should be able to be passed through a 10 mm port.

There were study limitations. The number of performed tests in this feasibility study was low and tests of devices were restricted to short term tests without measurement of duration of surgery or report of results of anastomosis. However, the self-locking device was intended for short term use only as the device with compressed tissue would be cut off at the completion of the anastomosis. Circular staplers are available in different sizes from several manufacturers. Our tests were limited to two models, which is a study limitation. However, the design of the device aimed to fit with any sized circular stapler, but additional tests are needed. In addition to circular staplers and CREX, other anastomosis techniques exist [19, 20]. Additional tests of the developed self-locking device throughout the available range of techniques of anastomosis and their different instrument sizes remain to be performed.

This study suggests improvements of minor details of the device. Future study proposals include objectively scoring its ease of use and studying if the device enables a shortened duration of surgery in comparison to existing techniques. Additionally, with low rectal anastomosis already being the technically most challenging [10, 21], the possibility of using the device in a narrow space should also be tested. The described device partly builds on a previously developed resorbable implant designed for ligation purposes [22–26]. The present device has also been subjected to initial tests in urology [27], aimed to apply the developed technology of resorbable polymers. The use of the self-locking device in the increasingly popular laparoscopic procedures, including robotic surgery, remains to be evaluated where comparisons to manual or mechanical purse-string suture [28] are close at hand.

## Conclusions

In summary, in this feasibility study the self-locking loop appeared to be a promising alternative to a traditional purse-string suture around anvil or trocar in colon anastomosis.

## Abbreviations

CREX: colorectal anastomosis; rejoin the intestine and validate the anastomosis; extract samples for analysis; x-ray through connected catheters
